# Viral genomes reveal patterns of the SARS-CoV-2 outbreak in Washington State

**DOI:** 10.1126/scitranslmed.abf0202

**Published:** 2021-05-03

**Authors:** Nicola F. Müller, Cassia Wagner, Chris D. Frazar, Pavitra Roychoudhury, Jover Lee, Louise H. Moncla, Benjamin Pelle, Matthew Richardson, Erica Ryke, Hong Xie, Lasata Shrestha, Amin Addetia, Victoria M. Rachleff, Nicole A. P. Lieberman, Meei-Li Huang, Romesh Gautom, Geoff Melly, Brian Hiatt, Philip Dykema, Amanda Adler, Elisabeth Brandstetter, Peter D. Han, Kairsten Fay, Misja Ilcisin, Kirsten Lacombe, Thomas R. Sibley, Melissa Truong, Caitlin R. Wolf, Michael Boeckh, Janet A. Englund, Michael Famulare, Barry R. Lutz, Mark J. Rieder, Matthew Thompson, Jeffrey S. Duchin, Lea M. Starita, Helen Y. Chu, Jay Shendure, Keith R. Jerome, Scott Lindquist, Alexander L. Greninger, Deborah A. Nickerson, Trevor Bedford

**Affiliations:** 1 Vaccine and Infectious Disease Division, Fred Hutchinson Cancer Research Center, Seattle, WA 98109, USA.; 2 Department of Genome Sciences, University of Washington, Seattle, WA 98195, USA.; 3 Department of Laboratory Medicine and Pathology, University of Washington, Seattle, WA 98195, USA.; 4 Washington State Department of Health, Shoreline, WA 98155, USA.; 5 Seattle Children’s Research Institute, Seattle, WA 98101, USA.; 6 Department of Medicine, Division of Allergy and Infectious Diseases, University of Washington, Seattle, WA 98195, USA.; 7 Brotman Baty Institute for Precision Medicine, Seattle, WA 98195, USA.; 8 Department of Pediatrics, University of Washington, Seattle, WA 98105, USA.; 9 Institute for Disease Modeling, Bellevue, WA 98105, USA.; 10 Department of Bioengineering, University of Washington, Seattle, WA 98105, USA.; 11 Department of Global Health, University of Washington, Seattle, WA 98195, USA.; 12 Department of Medicine, Division of Allergy and Infectious Diseases, University of Washington, Seattle, WA 98195, USA.; 13 Public Health - Seattle & King County, Seattle, WA98121, USA.; 14 Howard Hughes Medical Institute, Seattle, WA 98195, USA.

## Abstract

The rapid spread of severe acute respiratory syndrome coronavirus 2 (SARS-CoV-2) has gravely affected societies around the world. Outbreaks in different parts of the globe have been shaped by repeated introductions of new viral lineages and subsequent local transmission of those lineages. Here, we sequenced 3940 SARS-CoV-2 viral genomes from Washington State (USA) to characterize how the spread of SARS-CoV-2 in Washington State in early 2020 was shaped by differences in timing of mitigation strategies across counties and by repeated introductions of viral lineages into the state. In addition, we show that the increase in frequency of a potentially more transmissible viral variant (614G) over time can potentially be explained by regional mobility differences and multiple introductions of 614G but not the other variant (614D) into the state. At an individual level, we observed evidence of higher viral loads in patients infected with the 614G variant. However, using clinical records data, we did not find any evidence that the 614G variant affects clinical severity or patient outcomes. Overall, this suggests that with regard to D614G, the behavior of individuals has been more important in shaping the course of the pandemic in Washington State than this variant of the virus.

## Spiking variants

Understanding the impact of emerging SARS-CoV-2 variants is essential to inform the development of effective antiviral measures. Studies have indicated that the D614G substitution in the SARS-CoV-2 Spike protein may increase transmissibility, but to what extent this variant has propelled the pandemic is debated. Analyzing thousands of viral genomes together with clinical data, Müller *et al.* found no evidence that the 614G variant rose in frequency in Washington State mid-2020 due to increased transmissibility or virulence. Instead, the data provided a stronger signal that the surge in 614G was explainable by its repeated introduction into the state as well as local differences in lockdown measures—both human-catalyzed factors.

## Teaser

Initial SARS-CoV-2 dynamics in Washington State were driven by regional interventions and introductions of variants differing in viral loads.

## INTRODUCTION

After its emergence near the end of November or beginning of December 2019 in Wuhan, China, severe acute respiratory syndrome coronavirus 2 (SARS-CoV-2) rapidly spread around the world (*1*). In the United States, the first reported case of coronavirus disease 2019 (COVID-19), the disease caused by SARS-CoV-2, was found in Washington State on 19 January 2020 in a traveler who had returned from China 4 days earlier. Until the end of February, no additional cases of COVID-19 were reported in Washington State.

At the end of February, however, a case of COVID-19 was reported in Snohomish County, the same county where the initial case was reported. This case had no known travel history and constitutes the first reported case of community transmission in Washington State (*2*). Although genetically closely related to the initial case, the later sequenced cases share a common ancestor in early February and have been reported to likely be due to an independent introduction of the virus (*2*).

After these initial introductions, SARS-CoV-2 has been introduced repeatedly into Washington State from different parts of the globe. Viruses introduced later differed genetically from those introduced earlier, most notably in one amino acid in the spike protein that facilitates viral entry and includes the receptor-binding domain. Since its first occurrence, this amino acid substitution from aspartate (D) to glycine (G) at position 614 of the Spike protein increased in relative frequency around the world (visible at https://nextstrain.org/ncov/global?c=gt-S_614) and now represents the vast majority of all new cases of COVID-19 (*3*–*5*). This increase in relative frequency of the 614G variant has been proposed to be due to higher transmissibility of the 614G variant over the 614D variant (*4*, *6*). A modest increase in viral load has been observed in patients infected with the 614G variant (*4*, *7*). Recently, multiple in vitro studies in human cell lines found a three- to ninefold increase in infectivity of the 614G variant (*5*, *8*, *9*). However, it remains unclear whether these population-level trends are due to higher transmissibility of the virus or simply due to founder effects owing to strong bottlenecks when SARS-CoV-2 spread globally, as the D614G variant was introduced early on in the European COVID-19 epidemic and spread from Europe to the rest of the world.

Washington State differs regionally, from more densely populated areas at the coast to more sparsely populated areas inland. We here focused on differences between the spread on lineages of 614D and 614G in the context of regional differences within Washington State. Extensive local spread of SARS-CoV-2 was first detected in King County, which includes the city of Seattle. King County was also the first region in the state to take action to curb the spread of SARS-CoV-2, including several large companies in the area mandating work from home in early March 2020 (*10*). After a statewide lockdown, new cases began to fall in the whole state, except for Yakima County, where cases peaked substantially later than in the rest of the state.

Using viral genetic sequence data isolated from patients in Washington State between February and July 2020, we tested the impact of temporal differences in county level workplace mobility trends, as well as the role of introductions from outside the state in driving case loads. We additionally investigated potential transmissibility differences between the two spike variants by comparing viral loads using cycle thresholds for viral quantification. Last, we investigated whether the D614G amino acid substitution led to more severe disease in patients infected with SARS-CoV-2.

## RESULTS

### The Washington State outbreak was caused by repeated introductions and shaped by temporal differences in mobility reductions

**Fig. 1:**
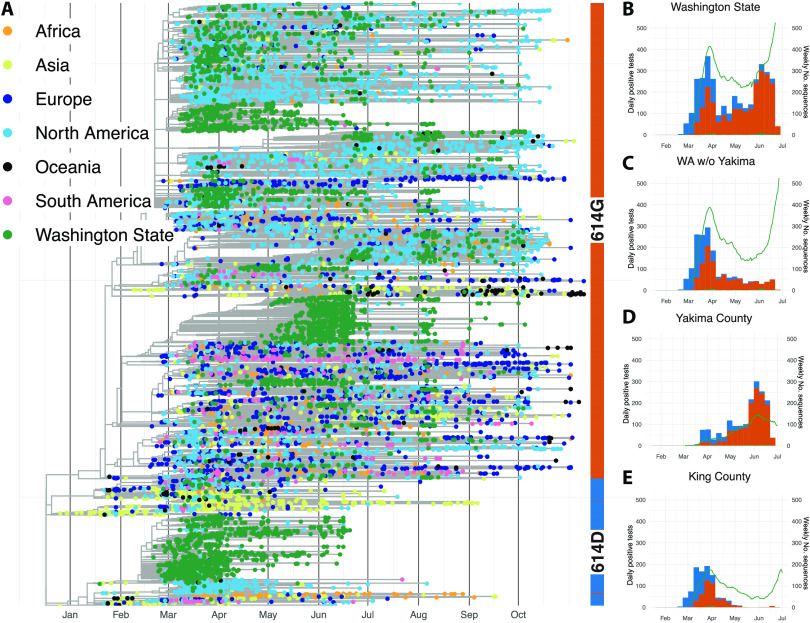
SARS-CoV-2 phylogeny highlighting D614G split and cases through time in Washington State. (**A**) Phylogenetic tree of 13,900 sequences from Washington State and around the world. Tips are colored on the basis of sampling location. This is a time-calibrated phylogeny with time shown in the *x* axis. The split between 614D sequences (blue) and 614G (orange) sequences is shown as a bar to the right of the phylogeny. (**B** to **E**) Confirmed cases and genetic makeup of SARS-CoV-2 across Washington State and individual counties. The green line shows a 7-day moving average of daily confirmed cases. The bar plots show weekly sequenced cases in our dataset. Cases due to the 614D variant are shown in blue, and cases due to the 614G variant are shown in orange. w/o, without.

We sequenced 3940 viruses from Washington State collected between February and July 2020 and used these sequences alongside other publicly available sequences from elsewhere in the world to characterize transmission dynamics. We observed that SARS-CoV-2 entered Washington State from different parts of the world and subsequently spread locally, evident as clusters of genetically similar Washington State viruses in the global phylogeny (Fig. 1A). In early February, an introduction of a 614D variant (*2*, *11*) fueled much of the early outbreak in March and April, but this lineage was supplanted through multiple introductions of 614G, and past April, the majority of viruses were 614G (Fig. 1).

To analyze the introduction and local spread of SARS-CoV-2 in Washington State, we first split these sequences into different local transmission clusters, which we defined as groups of sequences that originated from a single introduction into Washington State. To do so, we use a parsimony-based clustering approach, considering Washington State and everything outside Washington State as the two possible locations for parsimony clustering. The local transmission clusters obtained are shown at https://nextstrain.org/groups/blab/ncov/wa-phylodynamics?c=cluster_size, and their size distribution and D614G makeup are shown in fig. S1. We then used these local transmission clusters to analyze the spread of SARS-CoV-2 in the state using two phylodynamic approaches. First, we estimated the effective reproduction number (*R*_e_) using a birth-death approach (*12*), where we treated each individual local transmission cluster as independent observation of the same underlying population process (*13*). Next, we estimated effective population sizes over time and the degree of introductions using a coalescent skyline approach (*14*). To do so, we assumed that all sequences that clustered together were the result of local transmission and each individual cluster was the result of one introduction into Washington State. We then modeled the whole process as a structured coalescent process (*15*, *16*), where we assumed the migration history on the basis of the previous clustering (see Materials and Methods for details). In contrast to the birth-death model, the coalescent is conditioned on sampling, meaning that the information about population-level trends comes from the phylogenetic tree itself and not from the number of sequences through time.

**Fig. 2:**
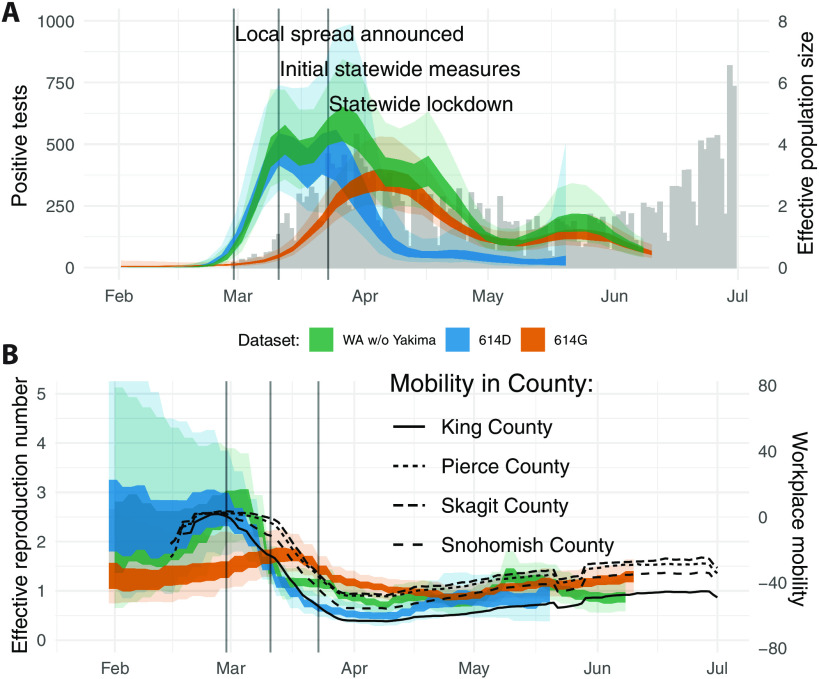
Regional dynamics of SARS-CoV-2 in Washington State inferred from confirmed cases and pathogen genomes. (**A**) Estimates of effective population sizes for the outbreak in Washington State (green interval) as well as for 614D (blue interval) and 614G (orange interval) individually as compared to confirmed cases in the state (gray bars). The inner band denotes the 50% highest posterior density (HPD) interval and the outer band denotes the 95% HPD interval. (**B**) *R*_e_ estimates using a birth-death approach for the same groups as in (A). The *R*_e_ estimates are compared to Google workplace mobility data for King, Pierce, Skagit, and Snohomish Counties shown as black solid and dashed lines. Workplace mobility is represented as a 7-day moving average.

We performed these phylodynamic analyses for a random subsample of 1500 samples from all Washington counties except for Yakima County as well as for the 614D (500 sequences) and 614G (1000 sequences) lineages separately. In addition, we performed the same analysis using 750 sequences from Yakima County only. After an initial introduction of SARS-CoV-2 (*2*), the number of cases grew rapidly (Fig. 2A). As expected, growth in confirmed cases was mirrored in phylodynamic estimates of viral effective population size (Fig. 2A). In addition, we observed maximal transmission intensity at the end of February 2020 when *R*_e_ was between 2 and 3 (Fig. 2B). This is consistent with other estimates of the effective reproduction number of SARS-CoV-2 during early phases of an epidemic when control measures are not in place (*17*–*19*).

Around the time when community spread in King County was announced on 29 February 2020, we observed decreased occupancy of workplaces according to Google mobility data (fig. S2) (*20*). This reduction in workplace mobility occurred relatively early in King County compared to other regions of the state that had little or no reported cases at the time (fig. S2). This is consistent with several businesses starting to institute measures, such as work-from-home policies, at the beginning of March (*10*). This reduction in mobility in King County coincided with a reduction in the effective reproduction number of 614D cases in the state (Fig. 2B). By the time initial statewide measures were implemented on the 11th of March, cases of 614D had almost peaked and were starting to decline, whereas overall cases were about constant or still increasing (Fig. 2A).

Cases of 614G were still increasing and peaked a little over a week later than cases of 614D (Figs. 1 and 2A). This was around the time when the statewide lockdown order came into effect on 24 March 2020. Whereas cases of 614D were initially mostly located around Seattle, cases of 614G were more widespread throughout the state. Viruses sampled from cases in Pierce County and in the counties north of King County mostly harbored the 614G variant (Fig. 1C). Changes in the effective reproduction number of 614G coincided with changes in mobility outside of King County (Fig. 2B). An alternative phylodynamic method using a coalescent approach yielded highly similar results (fig. S3).

Yakima County was the other county in the state besides King County with a large number of 614D cases later in the epidemic (Fig. 1D). The outbreak there happened later than the first large outbreak in King and neighboring counties. In addition, the trend in cases in Yakima County became increasingly decoupled from workplace mobility as measured by cellphone movement for reasons likely associated with a large population of essential workers in the agricultural sector and seasonal worker migration poorly captured in mobility metrics (fig. S4) (*21*, *22*).

To test whether amino acid substitutions beyond D614G affected the chance of SARS-CoV-2 of spreading locally, we next tested whether introductions of lineages with more amino acid substitutions were more successful in spreading locally. We computed the number of amino acid and nucleotide substitutions of the first sampled sequence of each local transmission cluster relative to Wuhan/Hu-1/2019 (*23*). We then estimated whether there was a relationship between the number of amino acid and nucleotide substitutions when a lineage was introduced into Washington State and whether that introduction was successful, which we defined as having led to detectable local transmission. Consistent with a previous publication (*24*), we did not find any substantial relationship between the number of amino acid substitutions and the success of an introduction (fig. S5).

Introductions of SARS-CoV-2 cases from different countries or different areas within a country have repeatedly been discussed as drivers of local outbreaks, particularly in the context of travel bans. We therefore investigated the importance of introductions in driving the outbreak in Washington State. We estimated the relative contribution of introductions compared to local transmission following the coalescent approach introduced above. In short, we used the estimated changes in effective population sizes over time and the estimated rates of introduction to compute the percentage of new cases in the state due to introductions (see Materials and Methods for details).

**Fig. 3:**
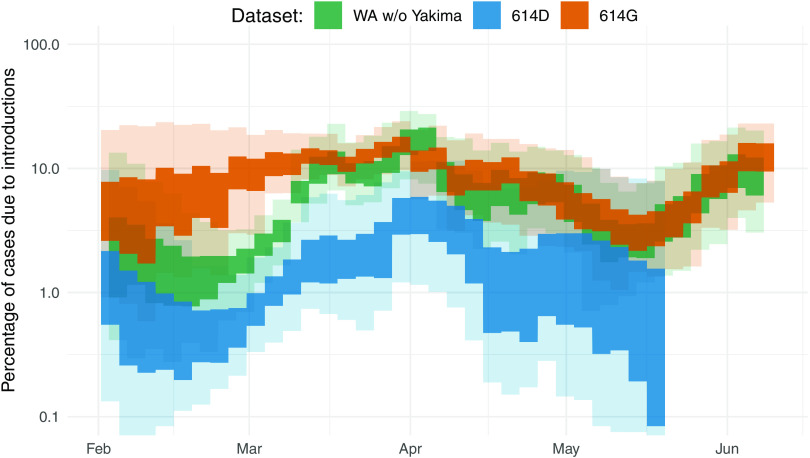
Phylogenetic estimate of the percentage of introductions of the overall cases. Percentages were estimated as the relative contribution of introductions to the overall number of infections using the multitree coalescent. Percentages are shown for the 2020 outbreak in Washington State (green interval) as well as for 614D (blue interval) and 614G (orange interval). The inner area denotes the 50% HPD interval and the outer area denotes the 95% HPD interval.

We estimated the percentage of new cases due to introductions in Washington State (excluding Yakima County) to be below 10% initially and to then have increased to about 10% by the middle of March through early April (Fig. 3). As a reference, the United States instituted a travel ban for nonresidents coming from China on 2 February 2020 and a travel ban from Europe effective 16 March 2020. Increases in the proportion of introductions of the overall cases can be driven by either a reduction in the local transmission rate or an increase in the rate of introduction.

The observed introductions were unevenly distributed across the different clades 614D and 614G (Fig. 3) (*6*, *25*). The proportion of introduced 614G cases was substantially greater than the proportion of introduced 614D cases. We estimated the percentage of introduced 614D cases to be below 3% during the whole outbreak. On the other hand, we inferred the percentage of introduced 614G cases to have been over 10% until the beginning of April. This means that a substantially higher fraction of 614G cases were caused by introductions than for 614D cases. This is expected, considering that cases of 614G were much more widespread outside of China (Fig. 1A), including in areas with relatively strong travel patterns to Washington State during the epidemic, such as New York State.

We next tested whether the percentage of new cases caused by introductions was reasonable given the number and size distribution of local transmission clusters. We simulated local transmission clusters where 0.1, 1, or 10% of all infections were caused by independent introductions. We found that the observed patterns in transmission cluster size distributions fell between the simulated patterns for 1 and 10% of all infections caused by recent introductions (fig. S6).

Overall, it appears that population-level changes in Washington State in relative frequencies of the two lineages can be explained by differences in timing of measures to curb the spread of SARS-CoV-2 on a county level and by repeated introductions of 614G. Although a parsimonious explanation of observed dynamics, this does not preclude 614G having a higher transmission rate relative to 614D. In addition, these population-level trends are affected by many confounding factors that are not directly related to the virus itself. We therefore next moved to investigate whether we could observe differences between individuals infected with viruses from either lineage on an individual level.

### D614G leads to higher viral load, without apparent effects on virulence

We tested for differences in viral loads between patients infected with either the 614D or the 614G viral variants by comparing cycle threshold (Ct) values. Ct values are inversely correlated with viral load, and differences in Ct values between these two variants have been reported previously (*4*, *7*). We analyzed 1743 sequenced SARS-CoV-2 samples from Washington State for which we had access to Ct values. We only used genomes sampled between February and April 2020, when both lineages were circulating in Washington State.

Of these 1743 genomes, 1128 genomes were from patients referred by a health care provider for nasopharyngeal swab testing to the University of Washington (UW) Virology laboratory. A total of 523 genomes were from samples collected by the Washington Department of Health (WA DOH), and 92 samples were from self-collected mid-turbinate nasal swabs mailed in for testing as part of the Seattle Coronavirus Assessment Network (SCAN). During this time period, UW Virology used multiple platforms for polymerase chain reaction (PCR) testing (fig. S7A). Because it is difficult to compare Ct values across primer sets and platforms (*26*), we mainly focused on samples amplified with the most common primer set: N1 and N2 (*n* = 879), although analyses using ORF1ab primers (*n* = 229) were also conducted.

**Fig. 4:**
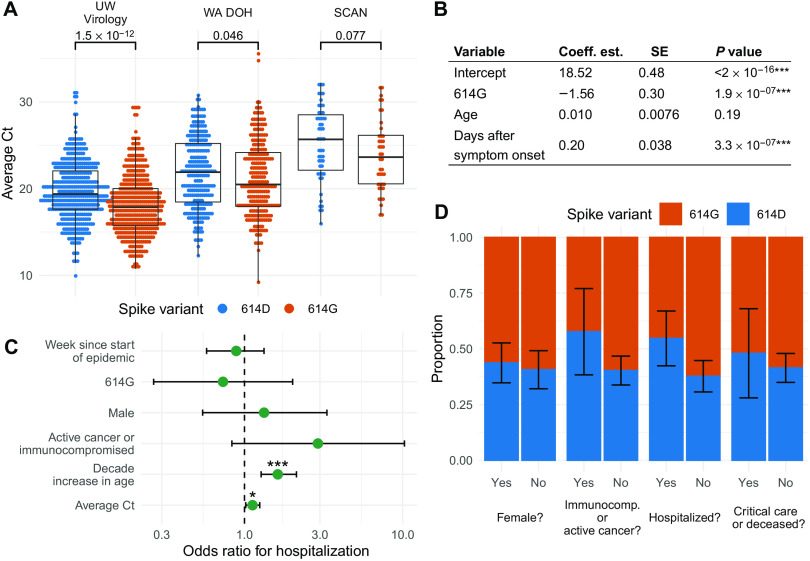
Factors affecting viral load and disease severity at an individual level. (**A**) Comparison between cycle threshold (Ct) values for viruses with 614G and 614D variants. (**B**) GLM analysis of Ct values using spike variant, age, and days since symptom onset as predictors. (**C**) Odds ratio of being hospitalized given infection with SARS-CoV-2. Error bars show 95% CI, corrected for multiple hypothesis testing using a Bonferroni correction. (**D**) Estimates of the average chance that a patient from a given group was infected with a virus from the 614D clade. The error bars denote the SE of the average chance that a patient from a group was infected with a virus from the 614D clade.

We found that patients infected with viruses with the 614G substitution had lower Ct values (higher viral load) than those infected with 614D viruses in all three collection channels (Fig. 4A and fig. S8). This difference was significant by Wilcoxon rank sum test in samples from UW Virology (N1 and N2 primers: median Δ = 1.5 cycles, *P* = 1.5 × 10^−12^; ORF1ab primers: median Δ = 2.5 cycles, *P* = 0.0012) and WA DOH (median Δ = 1.4 cycles, *P* = 0.046), but not in SCAN samples, where we had far fewer samples (median Δ = 2.1 cycles, *P* = 0.077) (Fig. 4A and fig. S8).

We next tested whether factors other than the D614G variant predicted Ct values. We applied a generalized linear model (GLM) assuming normally distributed Ct values to the UW Virology and SCAN samples using variant, patient age, and days after symptom onset as potential predictors of Ct values given that we, like others, have found Ct to be positively correlated with time since symptom onset (fig. S9A) (*27*–*30*). We found that the D614G variant and days since symptom onset were significant (*P* = 1.9 × 10^−7^) predictors of Ct values. Variant 614G has a Ct value that is, on average, 1.6 cycles lower than the 614D variant (N1 and N2 primers) (Fig. 4B) when controlling for age and time since symptom onset. This difference in Ct translated to a 0.47 log_10_ increase in viral load [95% confidence interval (CI): 0.29 to 0.64 log_10_], assuming the standard curve is linear in this region. For each day after symptom onset, Ct value was predicted to increase by 0.2 cycles (N1 and N2 primers: *P* = 3.3 × 10^−7^), which is consistent with other work on Ct values and infection time course (*27*–*30*). In SCAN samples, we observed similar coefficients and significance in the GLM (fig. S8). With ORF1ab primers, D61G variant was not a significant predictor; however, the residuals were not normally distributed, suggesting the model fit poorly with ORF1ab primers (fig. S8).

We additionally looked for a difference in time of symptom onset and sampling date between the two variants—sampling date could be a confounding variable because the relative abundance of the 614G variant increased over time (Fig. 1B)—but did not find any (fig. S9B). Because Ct values were shown to vary with effective reproduction numbers (*31*), we tested whether Ct values changed over time after accounting for the two spike variants. There were also no clear differences in Ct across time when accounting for the spike variant (fig. S9C).

We next tested whether substitutions other than spike D614G contributed to observed Ct differences. First, we considered the genetic diversity defined by five viral clades using the Nextstrain nomenclature: 19A, 19B, 20A, 20B, and 20C (fig. S10A). Clades 19B and 20C differed significantly in their Ct values from the other clades (mean Δ = 1.5 cycles, *P* adjusted ≤ 2 × 10^−8^ Tukey’s range test) (fig. S10B). However, when controlling for the 614G variant, clade membership was not predictive of Ct (fig. S10C). Most samples with available Ct fell into clades 19B and 20C, which primarily contained 614D and 614G variants, respectively, so there may not have been enough genetic diversity in our dataset to identify Ct differences with respect to the other viral clades.

Next, we explored the relationship between the number of amino acid substitutions different from Wuhan/Hu-1/2019 and Ct value. We did not find a significant correlation between amino acid substitutions and Ct with either of variants (614D: Pearson’s = −0.052, *P* = 0.14; 614G: Pearson’s = 0.061, *P* = 0.066) (fig. S11, A and B). However, a GLM of 614D variant samples predicted a 0.42 decrease in cycle threshold with each additional amino acid substitution (*P* = 0.011) (fig. S11C). In the same GLM applied to 614G variants, amino acid substitutions were not predictive of Ct (*P* = 0.66) (fig. S11D). Within the 614D variant, there was not a specific protein in which additional amino acid substitutions affected Ct values. This might suggest that within our dataset, 614G variants are at a local fitness maxima, whereas 614D variants are not. Thus, there could be more opportunity for amino acid substitutions in 614D variants to affect viral load. We may, however, miss some potentially confounding predictors in this analysis, such as age or mutations in a primer binding region, which could inflate the confidence in the results.

We also found a difference in the age of people infected between the two lineages (fig. S12). In samples from UW Virology, the average age of patients infected with viruses from the 614D and 614G lineages was 56.6 and 52.4, respectively (*P* = 5.8 × 10^−4^, Student’s *t* test). In SCAN samples, the average age of patients was 45.8 for 614D and 38.4 for 614G (*P* = 0.088). Age differences may be caused by increased testing, resulting in detection of less severe, younger cases later in the epidemic when 614G was more prevalent. However, we tested this hypothesis in a GLM with week of sample collection and D614G variant as potential predictors of age. Individuals with 614G variant were 3.5 years younger on average (*P* = 0.0098), whereas sample week was not a significant predictor of age (*P* = 0.20) (fig. S12). A skew toward younger individuals is consistent either with a more transmissible virus or with more severe infection as this would result in a larger fraction of younger patients seeking testing. However, the absolute difference in age of infection was still small.

We had access to additional clinical information for 248 of the 1128 sequences from patients referred for SARS-CoV-2 testing by a health care provider. One hundred four of these patients were infected with viruses from the 614D clade, and 144 patients were infected with viruses from the 614G clade. We used data from electronic health records to examine whether differences in Ct values held after correcting for additional potentially confounding factors. We performed the same GLM analysis as above but omitted days since symptom onset as it was missing from most samples. We included additional potential predictors, such as sex, active cancer or immunocompromised status, hospitalization, and whether a patient required intensive care or died. We again found the D614G variant to be significantly associated with Ct values (N1 and N2 primers, *n* = 184, *P* = 0.03). Sex was also a significant predictor of Ct with male individuals having Ct values 1.09 units lower than female individuals (SE = 0.48, *P* = 0.02). None of the other predictors were found to be significant in predicting Ct values, which might be driven by small sample size (table S1). With ORF1ab primers, the D614G variant was not significantly associated with Ct values nor were residuals normally distributed (*n* = 63) (table S2). ORF1ab primers were used later in the epidemic when the 614D variant was less abundant (fig. S7B).

We next investigated which factors associated with clinical outcome. We grouped cases into inpatient (hospitalized) or outpatient (not hospitalized) and then performed a logistic regression with inpatient or outpatient as potential outcomes. As factors predicting outcome, we considered clade membership, sex, immunocompromised/active cancer, age, week of testing, and measured Ct value. Age (*P* = 3.2 × 10^−6^) and measured Ct value (*P* = 0.012) were significant predictors for hospitalization after Bonferroni correction for multiple hypothesis testing. Whether a patient was suffering from active cancer or was immunocompromised had an estimated odds ratio of 2.9 (0.8 to 10.8) but was not significant (*P* = 0.14). We did not find any evidence that D614G variant affected clinical outcome (Fig. 4C). This is consistent with neither variant being significantly enriched among males, immunocompromised/active cancer patients, hospitalized patients, or patients who required intensive care or succumbed due to COVID-19 (Fig. 4D).

## DISCUSSION

The COVID-19 pandemic has greatly affected lives around the world. As a virus that just recently made the jump into humans, understanding its transmission dynamics and the drivers of its spread is of utmost importance. The emergence of more transmissible strains of SARS-CoV-2 based on an increase in relative frequencies over time has been suggested previously (*25*).

Consistent with trends from other locations around the world (*4*), we found that cases of the spike 614D variant initially dominated in Washington State but were later taken over by spike 614G. However, the trends for 614G and 614D cases that we observed in Washington State appeared to be explained by differences in when action to curb the spread of SARS-CoV-2 were taken on a county level. The trends in effective reproduction numbers between the two clades 614G and 614D coincided with the different trends in mobility of King County (which includes Seattle) and other areas that experienced substantial spread of SARS-CoV-2. The observed patterns are consistent with initial spread of the 614D clade being largely concentrated in King County, which was then mitigated early on. Spread of 614G on the other hand, although present in King County, dominated in other areas of the state and the reduction in the *R*_e_ of this variant coincided with a reduction in mobility in these areas, which happened about 9 days after King County. The spread of SARS-CoV-2 in Yakima County, however, seems to be poorly captured by mobility trends.

We additionally inferred introductions play a larger role in driving cases of the 614D variant than of the 614G variant. This suggests that differences in the relative frequencies of the two variants are at least, in part, driven by differences in when and where lineages were introduced into the state. Overall, we find that we can explain the changes in relative frequency of the 614D and 614G variants over time by nonviral factors in absence of intrinsic transmission rate differences. This does, however, not exclude the possibility that such differences exist and have led to the replacement of 614D by 614G in other parts of the world. The observation that changes in patterns of which lineages are introduced into a location can drive changes in local frequencies of a variant is important when evaluating whether new variants (such as B.1.1.7) are more transmissible. In particular, it means that an increase in relative frequency of a new variant in different places does not necessarily provide independent evidence about whether or not the new variant is more transmissible.

We did find evidence for lower Ct values in patients infected with viruses of the 614G variant, suggesting higher viral loads. This holds even after including several additional factors, such as the age of a patient and days since symptom onset, as potential predictors for Ct values. However, we did not find evidence that D614G has an impact on risk of hospitalization although testing policy would bias toward finding a variant with greater virulence as hospitalized patients are overrepresented in the dataset (*32*, *33*). The differences in Ct values translate to an about 0.47 log_10_ increase in viral load (95% CI: 0.29 to 0.64 log_10_). This difference might not be large enough to lead to large differences in severity or transmissibility that can be observed in a dataset of this size.

Our findings are broadly consistent with other analyses on the spike D614G substitution. A previous study found evidence of lowered Ct but limited clinical difference for viruses of the 614G clade in Sheffield, UK (*4*). Recent in vitro studies showed that pseudovirus containing spike protein with a 614G substitution exhibits greater infectivity (*5*, *8*, *9*). Other work suggests the increased transmissibility of 614G over 614D in an analysis of thousands of sequences from the United Kingdom (*6*).

Although our results are broadly consistent with other analyses, they are not without limitations. First, the sample collection is likely biased toward more symptomatic cases. In addition, the collection of SARS-CoV-2 samples was limited initially and improved during the study period and likely differed across different geographic areas. In other words, the sampling regime likely differed across space and time, potentially affecting the results.

The phylogenetic analyses conditioned on specific clustering of sequences in Washington State by incorporating background sequences from other locations. Differences in sampling and sequencing regimes in potential source locations of SARS-CoV-2 relative to Washington State could bias this clustering, which, in turn, could affect the estimated rates of introductions into Washington State and potentially also the effective reproduction numbers over time. Last, the phylodynamic methods used here make a few simplifying assumptions about how SARS-CoV-2 is spread, such as random sampling of infected individuals, homogeneous mixing of individuals, or the absence of superspreading. Although we addressed the latter in our simulation study, it is not fully clear how some of these simplifying assumptions affected the inference results.

Overall, we found evidence for higher viral loads in individuals with viruses from the 614G clade, which theoretically could affect transmissibility and severity. However, we did not see strong evidence that this degree of difference in Ct manifested in substantial differences in transmissibility or severity of infection with SARS-CoV-2 in the spring/summer 2020 Washington State epidemic.

## MATERIALS AND METHODS

### Study design

The aim of this study was to characterize the drivers of the SARS-CoV-2 outbreak in Washington State (USA) over several months and to investigate how different viral variants affected the spread of SARS-CoV-2. We collected genetic sequence data from SARS-COV-2 viruses isolated in Washington State. Here, we analyzed 3940 SARS-CoV-2 genomes sequenced from samples collected in Washington State between February and July 2020 as our primary dataset. These sequences were produced as part of an effort to survey the spread and evolution of SARS-CoV-2 in the state. These samples were pooled from three different channels: UW Virology, WA DOH, and SCAN, as described below.

Sequencing and analysis of samples from the Seattle Flu Study was approved by the Institutional Review Board (IRB) at the University of Washington (protocol STUDY00006181). Informed consent was obtained for all community participant samples and survey data. Informed consent for residual sample and clinical data collection was waived. Sequencing and analysis of samples from SCAN was approved by the IRB at the University of Washington (protocol STUDY00010432). Informed consent was obtained for all community participant samples and survey data. For UW Virology Lab, use of residual clinical specimens was approved by the IRB at the University of Washington (protocol STUDY00000408) with a waiver of informed consent.

### Sample collection and testing for SARS-CoV-2

For the 1236 UW Virology samples, nasopharyngeal/oropharyngeal swabs were obtained as part of clinical testing for SARS-CoV-2 ordered by local health care providers or collected at drive-up testing sites. RNA was extracted and the presence of SARS-CoV-2 was detected by reverse transcription–PCR (RT-PCR) as previously described using either the emergency use-authorized UW Centers for Disease Control (CDC)–based laboratory-developed test, Hologic Panther Fusion test, or Roche cobas SARS-CoV-2 test (*34*).

For the 2601 WA DOH samples, nasopharyngeal/oropharyngeal/bronchoalveolar/sputum samples were obtained for SARS-CoV-2 clinical testing, as requested by submitting health care entities. RNA was extracted and the presence of SARS-CoV-2 was detected via either the CDC 2019-nCoV RT-PCR Diagnostic Panel or the Applied Biosystems TaqPath COVID-19 Combo Kit.

For the 103 SCAN samples, specimens were shipped to the Brotman Baty Institute for Precision Medicine via commercial couriers or the U.S. Postal Service at ambient temperatures and opened in a class II biological safety cabinet in a biosafety level-2 laboratory. Two or three 650-μl aliquots of Universal Transport Media were collected from each specimen and stored at 4°C until the time of nucleic acid extraction, performed with a MagNA Pure 96 small volume total nucleic acid kit (Roche). SARS-CoV-2 detection was performed using real-time RT-PCR with a probe set targeting Orf1b and S with FAM fluor (Life Technologies 4332079, assay nos. APGZJKF and APXGVC4APX) multiplexed with an RNaseP probe set with VIC or HEX fluor (Life Technologies A30064 or IDT custom) each in duplicate on a QuantStudio 6 instrument (Applied Biosystems).

### Viral sequencing and genome assembly

For UW Virology samples, sequencing was attempted on all specimens with Ct < 32 either using a metagenomic approach described previously (*2*, *35*), via oligonucleotide probe-capture (*36*), or using an amplicon sequencing–based approach (*37*). Libraries were sequenced on Illumina MiSeq or NextSeq instruments using 1 × 185 or 1 × 75 runs, respectively. Consensus sequences were assembled using a custom bioinformatics pipeline (https://doi.org/10.5281/zenodo.4701603) that combines de novo assembly and read mapping to generate a per-sample consensus sequence. Consensus sequences were deposited to GenBank and GISAID and raw reads to SRA under Bioproject PRJNA610428.

For samples from WA DOH and SCAN, sequencing was attempted on all specimens with Ct < 30 using a hybrid-capture approach. RNA was fragmented and converted to cDNA using random hexamers and reverse transcriptase (SuperScript IV, Thermo Fisher Scientific) and a sequencing library was constructed using an Illumina TruSeq RNA Library Prep for Enrichment kit. Using Ct value as a proxy for viral load, samples were balanced and pooled in 24-plex for the hybrid capture reaction. Capture pools were incubated overnight with probes targeting the Wuhan-Hu-1 isolate, synthesized by Twist Bioscience. The manufacturer’s protocol was followed for the hybrid capture reaction and target enrichment washes. Final pools were sequenced on the Illumina NextSeq or NovaSeq instrument using 2 × 150–base pair reads. The resulting reads were assembled against the SARS-CoV-2 reference genome Wuhan/Hu-1/2019 (GenBank accession MN908947) using the bioinformatics pipeline at (https://doi.org/10.5281/zenodo.4701970). Consensus sequences were deposited to GenBank and GISAID. Samples sequenced by UW Virology had a higher proportion of 614G variants (54.7%) than SCAN and WA DOH samples (48.6%), which were sequenced using a different pipeline (chi-square test: *P* = 0.017). Investigating differences in Ct independently for each primer type should control for differences in the spike variant proportion, as primer types did not overlap between sequencing pipelines.

### Clustering

To distinguish between sequences that were connected by local transmission, we clustered all sequences from Washington State together on the basis of their pairwise genetic distance. We first built a timed tree using sequences from Washington State and from around the world using the Nextstrain pipeline (*3*). Overall, we used 4023 sequences from Washington State and 6028 from the rest of the world. Of all sequences, 2601 were from the Washington Department of Health, 1236 were from the UW Virology Lab, and 103 were from SCAN. All other sequences were downloaded from the GISAID EpiCoV database (*38*, *39*).

We then use a parsimony-based approach to reconstruct the locations of internal nodes. We considered all sequences from Washington State as one location and all sequences from anywhere else on the globe to be from another location. We then reconstructed the internal node locations using the Fitch parsimony algorithm. We considered each group of sequences to be on the same local transmission cluster if all their common ancestor nodes are inferred to be in Washington State. We additionally tested the sensitivity of this approach to having less background samples by randomly removing sequences from outside of Washington State and computing the number of clusters again. Although we do expect that including more background sequences would increase the number of clusters detected, we did not find a large impact on the number of background sequences on the number of clusters identified or the average size of clusters identified (fig. S13).

### Estimating population dynamics jointly from multiple local outbreak clusters

To estimate the population dynamics of the Washington State outbreak, we used a coalescent approach to infer these dynamics jointly from all known local outbreak clusters. We modeled the coalescence and migration of lineages within Washington State as a structured coalescent process with known migration history. Under this model, lineages can coalesce within the sampled subpopulation and have originated from outside the sampled subpopulation. We a priori assumed that we know where on the tree lineages were introduced into the sampled subpopulation (fig. S14). This known migration history is given by the clustering of sequences into local outbreak clusters. The migration events from anywhere outside WA into WA were always assumed to have happened before the common ancestor of all sequences in each local outbreak cluster. How long before this common ancestor time was inferred during the Markov chain Monte Carlo (MCMC) run. The rate at which we expect coalescent events to occur is exponentially distributed with mean = *n* × (*n* − 1)/2*N*_e_, and the rate at which we expect to observe introductions events is exponentially distributed with mean *n* × *m*, with *n* being the number of lineages in any given local transmission cluster that coexist at a point in time and *m* being the rate of introduction. Everything that happened outside the sampled subpopulation was ignored, or in other words, we ignored how exactly the individual local outbreak clusters related to each other.

We then inferred the effective population size and rates of introduction through time using a skyline approach. Effective population sizes and rates of introduction were allowed to change at predefined time points. The rates were interpolated between these predefined time points where the rates are estimated. This is equivalent to assuming exponential growth or decline between the effective population sizes at these time points.

We then used two different ways to account for correlations between adjacent scaled effective population sizes (*N*_e_τ). First, we used the classic skyride (*14*) approach, where we assumed that the logarithm of adjacent *N*_e_τ is normally distributed with mean 0 and an estimated σ. In addition, we used an approach where we assumed that differences in growth rates are normally distributed with mean 0 and an estimated σ (*40*). This is equivalent to using an exponential coalescent model with time varying growth rates. We implemented this multitree coalescent approach as an extension to the Bayesian phylogenetics software BEAST2 (*41*). The code for the multitree coalescent is available here (https://doi.org/10.5281/zenodo.4697903) and is validated in fig. S3. We allowed the effective population sizes to change every 3.5 days and the rates of introduction to change every 7 days. The inference of the effective population sizes and rates of introductions was performed using an adaptive multivariate Gaussian operator (*42*) implemented at (https://doi.org/10.5281/zenodo.4705996), and the analyses were run using adaptive Metropolis-coupled MCMC (*43*).

In contrast to backward-in-time coalescent approaches, we can consider different local outbreak clusters as independent observations of the same underlying population process using birth-death models. We inferred the effective reproduction number using the birth-death skyline model (*12*) by assuming that the different local outbreak clusters are independent observations of the same process with the same parameters (*13*). We allowed the effective reproduction number to change every 3.5 days. As for the coalescent approach, we assumed adjacent effective reproduction numbers to be normally distributed in log space with mean 0 and an estimated σ. We further assumed the becoming uninfectious rate to be 52.3 per year, which corresponds to an average duration of infectivity of 7 days (*44*). We allowed the probability of an individual to be sampled and sequenced upon recovery to change every 7 days.

### Simulation study

To test our implementation of the multitree coalescent, we performed two different sets of simulation studies. In the first simulation study, we simulated 10 phylogenetic trees under the structured coalescent using 1000 samples from the same location in MASTER (*45*). For each of the 10 simulations, we randomly sampled the *N*_e_ at time 0 from a normal distribution with mean = 0 and σ = 0.5 and then randomly drew the *N*_e_ at subsequent time points *t* + 1 randomly from a normal distribution with mean = *N*_e_(*t*) and σ = 0.5. This is equivalent to randomly sampling *N*_e_ trajectories under a skygrid distribution (*14*). We performed the same for the rate of introductions at different points in time. We then simulated a single phylogenetic tree under the structured coalescent using these randomly sampled parameters. Next, we splitted this tree into several local transmission clusters and then inferred the *N*_e_s and rate of introductions over time from only the local transmission clusters (fig. S15).

In the second simulation study, we simulated 10 phylogenetic trees under a structured infected (I) only model with superspreading. We assumed that there was a constant number of introductions per unit of time from outside into Washington State. After an introduction into the state, each infected individual was transmitting to *n* other individuals. We assumed the number of newly infected individuals to be negatively binomially distributed such that the mean number of introductions at any point in time *t* was equal to *R*_e_(*t*) and the dispersion parameter *k* = 1. We next simulated a structured phylogenetic tree from this approach. We then simulated genetic sequences on top of this phylogenetic tree using Seq-Gen (*46*).

### Subsampling of sequences

We analyzed the population dynamics in total for four different datasets. In the first datasets, we randomly subsampled 1500 of the sequences from Washington State, excluding sequences from Yakima County. One thousand five hundred sequences were chosen because of computational limitations of the Bayesian phylodynamic inference. For the second and third datasets, we distinguished between two different clades that we call D and G. The D clade consists of all sequences with an aspartic acid at site 614 of the spike protein, and the G clade consists of all sequences with a glycine at this position (visible at https://nextstrain.org/ncov/global?c=gt-S_614). For the 614D datasets, we used the same subsampling procedure as for the above dataset but with 500 sequences, and 750 sequences for the 614G clade. For the Yakima County dataset, we used 750 randomly subsampled sequences.

### Estimating the percentage of overall new cases from independent introductions

We estimated the relative contribution of introductions compared to local transmission using the coalescent approach introduced here. In addition to the regular assumptions of the coalescent approach that all samples are taken at random from a well-mixed population, we assumed that differences in effective population size between adjacent time intervals can be used to compute the transmission rate. We then computed the transmission rate as the sum of the growth rate of the effective population size and the becoming uninfectious rate (that is, we used the relationship $\frac{\mathrm{dNe}}{\mathrm{dt}}=\text{transmission rate}-\text{becoming uninfectious rate}$, to compute the transmission rate). We assumed an average time of infectiousness of 7 days. In addition, we assumed that *dN*_e_/*dt* is independent from the rate of introduction. We then computed the percentage of introductions in overall cases using the rate of introduction and the transmission rate. The rate of introduction can be expressed as the total number of introductions divided by the number of infected in WA, that is, rate of introduction = No. introductions/No. infected. The total number of new infections locally can be expressed as transmission rate × infected, which, in turn, means that ratio of introductions over local infections can be expressed as (rate of introduction × infected)/(transmission rate × infected). From this ratio, we can then compute the percentage of introductions of the overall cases.

We tested that we can retrieve the percentage of introductions from simulations, where we simulated phylogenetic trees using an infected recovered (IR) compartmental model with superspreading using MASTER (*45*). We then simulated genetic sequence data using those trees and then inferred the percentage of new cases due to introductions from those sequences (figs. S15 and S16).

### Chart review

Clinical record review of UW affiliated patients was performed under University of Washington IRB: STUDY00000408. This included patients who visited UW affiliated clinics and patients who were hospitalized at UW Medical Center, both the Montlake and Northwest locations, and Harborview Medical Center. Sex, age, the presence of active cancer or immunosuppresive medication, hospital admission, critical care admission, and deceased status were extracted from all charts.

### Statistical analysis

#### Factors affecting Ct and clinical outcomes of individuals

R/3.6.2 was used for Ct and clinical record analysis. The code and data cleaned of all patient identifiers is available at (https://doi.org/10.5281/zenodo.4701583).

UW Virology used three different primer sets and platforms over the timeframe of the dataset (fig. S7). Because it is difficult to compare Ct across primer sets, we ran both tests comparing Ct by viral clade and the generalized linear model predicting Ct separately for N1 and N2, and ORF1ab primers. There were insufficient samples amplified with Egene/RdRp primers for statistical analysis (*n* = 20).

We chose to use Wilcoxon rank sum test to compare differences in Ct between viral lineages and Student’s *t* test to compare differences in age between viral lineages. Age was reported as a decade bin converted into a numerical equivalent, and Wilcoxon rank sum test underestimates differences with duplicate numbers. Tukey’s range test was used to identify differences in Ct between viral clades, and we used Pearson’s correlation coefficient to examine the relationship between Ct and number of amino acid and synonymous substitutions. *P* values less than 0.05 were considered significant. Data were plotted as a univariate histogram to check for normal distribution before testing with Tukey’s range test and Student’s *t* test.

For GLMs predicting Ct and age, we used a multivariate linear regression of form

$$y_{i}=\beta_{0}+\Sigma \;\beta_{j}x_{i,j}+\epsilon_{i}$$

where *y* is the dependent variable (either Ct or age), β is the coefficient of the predictor variable, *x* is the predictor variable, and ϵ is the residual error. Models were run with the glm package in R.

UW Virology and SCAN samples were used to estimate predictors of Ct as age was not available for WA DOH samples. The predictor variables were the amino acid at Spike 614 (binary variable), days since symptom onset (continuous variable), and age of patient (continuous variable). In the GLM of Ct with only samples from UW Medicine affiliates, we excluded days since symptom onset as it was not available for most samples. We additionally included sex (binary variable), active cancer or immunocompromised (binary variable), hospitalized (binary variable), and required critical care or deceased (binary variable) as predictors of Ct. When considering viral clade as a predictor of Ct, we applied the same GLM as above with addition of binary variables for clade 19A, 20A, and 20C. Clades 20B and 19B were excluded due to collinearity.

To test the relationship between number of substitutions (synonymous and amino acid) and Ct, we applied a GLM predicting Ct from amino acid substitutions (continuous variable), synonymous substitutions (continuous variable), days since symptom onset (continuous variable, week since start of the Washington State epidemic (continuous variable), and binary variables for ORF1ab, WA DOH, and SCAN primers. We ran the GLM separately spike 614D and 614G variants as the correlation between the number of amino acid substitutions and Ct differed between variants. In the GLM, we excluded samples with greater than 20 nucleotide substitutions as outliers, because all other samples had between 3 and 17 nucleotide substitutions.

To estimate predictors of patient age, we used all SCAN & UW Virology samples with age available (*n* = 1172). The predictor variables were amino acid at spike 614 (binary variable) and week since community spread of COVID-19 was reported in Washington (continuous variable).

To estimate predictors of hospitalization if infected with SARS-CoV-2, we used a multivariate logistic regression

$$\text{logit}(P_{i})=\beta_{0}+\Sigma \;\beta_{j}\;x_{i,j}+\epsilon_{i}$$

where *P* is the probability of hospitalization, β is the coefficient of the predictor variable, *x* is the predictor variable, and 𝜖 is the residual error. Predictor variables were week since first sample in dataset (continuous variable), sex (binary variable), active cancer or immunocompromised (binary variable), age in decade (continuous variable), amino acid at Spike 614 (binary variable), and average Ct (continuous variable). To fit the logistic regression, we again used the glm package in R, specifying family as “binomial”. *P* values and CIs for risk of hospitalization were adjusted for multiple hypothesis testing using a Bonferroni correction.

Chi-square tests were used to compare proportions of viral lineages by sex, immunocompromised status, clinical outcome (inpatient or outpatient), and severe outcome (critical care or death). *P* values were adjusted for multiple hypothesis testing using the Bonferroni correction.

## SUPPLEMENTARY MATERIALS

stm.sciencemag.org/cgi/content/full/scitranslmed.abf0202/DC1 Fig. S1. Number of lineages through time for different local transmission clusters. Fig. S2. Workplace mobility trends of different counties in Washington State compared to King County. Fig. S3. *R*_e_ estimates using the coalescent skygrowth model compared to Google mobility data. Fig. S4. Effective reproduction number and workplace mobility in Yakima County. Fig. S5. Substitutions and success of a SARS-CoV-2 introduction. Fig. S6. Probability that a newly sampled case reveals a new introduction. Fig. S7. Histogram of primers used by UW Virology across time. Fig. S8. Comparison of Ct across SARS-CoV-2 Spike variant. Fig. S9. Symptom and Ct values across time. Fig. S10. Comparing Ct by viral clade. Fig. S11. Cycle threshold by number of substitutions. Fig. S12. Age of infected individuals by 614D or 614G variant over time. Fig. S13. Dependence of the local outbreak clusters and the number of background sequences used. Fig. S14. Principle of the multitree coalescent. Fig. S15. Estimation of effective population sizes and rates of introductions from simulations. Fig. S16. Estimation of the percentage of new cases due to introductions from simulations. Table S1. GLM of Ct with N1, N2 primers in patients at UW affiliates. Table S2. GLM of Ct with ORF1ab primers in patients at UW affiliates. Data file S1. GISAID acknowledgment table (tsv). Data file S2. Cycle threshold values for isolates (tsv).

## Appendix

View/request a protocol for this paper from *Bio-protocol*.
